# Editorial: Antimicrobial Peptides and Complement – Maximising the Inflammatory Response

**DOI:** 10.3389/fimmu.2015.00491

**Published:** 2015-09-24

**Authors:** Cordula M. Stover

**Affiliations:** ^1^Department of Infection, Immunity and Inflammation, College of Medicine, Biological Sciences and Psychology, University of Leicester, Leicester, UK

**Keywords:** inflammation, complement, antimicrobial peptides, immune response, inflammatory response

Striking commonalities in the roles of complement and antimicrobial peptides have recently been reported; their abilities to apply selection pressures on a bacterial population in the bloodstream (1), to contribute to enhanced phagocytosis of opsonized bacteria (2), and to interactively determine skin microbiome (3). Evolutionary roots for complement proteins and antimicrobial peptides are ancient (4). Predating the avenue of somatic recombination, antimicrobial peptides and complement have further emerged as modulators of cell activities that are part of the adaptive immune response. Therefore, antimicrobial peptides and complement were logical contenders for a focused analysis to distil from a wide complexity a range of overlapping and distinct activities that could serve to maximize local and systemic inflammatory responses.

The task was ambitious. Aiming to draw together experts and junior scientists in two distinct areas of inflammatory responses, an e-book series was produced which in its entirety challenges oligofactorial analyses in health and disease and points to a gain in embracing more fully the interconnection of inflammatory reactions and their components using, as examples, complement and antimicrobial peptides.

Functional analytical approaches may be derived from genomic analyses using cross species comparisons for antimicrobial peptides and is demonstrated in Machado and Ottolini’s article (5). Significant copy number variations for defensin genes in and between populations make these exciting modulators of inflammation, mucosal immunity, and infection responses. In the complement system, gene duplication leading to C4A and C4B and functional polymorphisms in the MBL gene (in humans) provide variability in the fluid phase of complement activation.

In two parts, Roumenina’s team provide a delicately researched state-of-the-art evaluation of complement activation and its regulation as well as a summary of current understanding of the mutlifaceted roles of complement anaphylatoxins in inflammation (6, 7).

Bevington’s group put forward a case in support of further avenues of research to identify pH sensing molecules and understand pH-dependent contact and complement system activation and their interactions (8). The activity of antimicrobial peptides is also influenced by pH conditions (9). It appears therefore that more rigorous measurements of pH in *in vivo* models may help to discern a level of regulation that is currently still underappreciated.

Day and Clark’s group remind that sialic acids or glycosaminoglycan structures on the cell surface or in the extracellular compartment provide interfaces, which can determine propagation or inhibition of complement activation and be the basis for tissue-specific susceptibilities to targeting binding of complement proteins (10). Interestingly, in *Drosophila*, engagement of the sulfated polysaccharide chains of heparin sulfate proteoglycans leads to expression of antimicrobial peptides (11).

Stadnyk’s group presents a program of work to test hypotheses or inferred models of interaction, which are relevant in understanding pathomechanisms of colitis, but also contribute to our understanding of mucosal tolerance (12). Much has yet to be gained from studying the luminal role of antimicrobial peptides vs. the mucosal role of complement to maintain the mucosal barrier (13).

In the periodontal pocket, *Porphyromonas gingivalis*, its reciprocal interaction with complement and antimicrobial peptides during periodontitis is associated with altered local microbiota, bone loss, and evasion to atherosclerotic plaque (14). Chemokines, for which direct antimicrobial activities have been shown, are the focus in Sahingur and Yeudall’s treatise on molecular determinants in the development and progression of oral cavity cancers. Produced in response to a polymicrobial insult, locally produced chemokines are relevant to epithelial dysplasia and osteoclast activity and, furthermore, shape the tumor microenvironment (15). Al-Rayahi and Sanyi juxtapose complex activities of antimicrobial peptides and complement components in a wide range of cancers and remind us of early tumoricidal work using bacterial extracts (16).

Rocha-Ferreira and Hristova discuss for the neonatal brain the role of complement and antimicrobial peptides in the dynamics and extent of inflammation and their potential as targetable mediators of hypoxia-induced brain damage (17).

A cautionary tale is told by Schuerholz et al. and Thompson et al., who deal with antimicrobial peptides and complement, respectively, in human sepsis (18, 19). Interactions of host to pathogen are multimodal and immune markers alter over the duration of disease. It seems reasonable to propose that parallel measurement of humorally accessible complement and antimicrobial peptides, players of the innate immunity bridging the adaptive immunity, will yield greater understanding of the dynamic host response during sepsis.

Finally, Zimmer et al. systematically present activity signatures of complement and antimicrobial peptides in homeostasis and disease (20) and point to a need to distinguish other activities, which relate to routine design of recombinant protein expression (21).

In their entirety, the contributions, by providing succinct and critical summaries, primary data and viewpoints, achieve to deepen insight in and understanding of complex matters involving and surrounding antimicrobial peptides and complement. The mind may become more prepared to consider a multipronged approach to health and disease, impacting on both, experimental and therapeutic designs.

## Conflict of Interest Statement

The author declares that the research was conducted in the absence of any commercial or financial relationships that could be construed as a potential conflict of interest.
